# Electronic health record–based identification of inpatients receiving antibiotic treatment for community-acquired pneumonia

**DOI:** 10.1017/ash.2023.253

**Published:** 2023-09-29

**Authors:** David Yang, Leigh Cressman, Keith Hamilton, Lauren Dutcher

## Abstract

**Background:** Inappropriate antibiotic use for community-acquired pneumonia (CAP) is common. Although antibiotic stewardship activities require real-time, accurate identification of patients being treated for CAP, there are few reliable methods to identify such patients using the electronic health record (EHR). We conducted a retrospective study to assess the performance of provider-selected antibiotic indication in identifying patients being treated for CAP among a cohort of hospitalized adults. **Methods:** We randomly selected 440 patients from a cohort of patients who received at least 1 systemic antibiotic within 48 hours of admission between January 1, 2019, and December 31, 2021, at 3 acute-care hospitals. The reference standard for treatment of CAP was defined as intention to treat for pneumonia by inpatient provider(s) within 48 hours of admission, as assessed by chart review of provider notes. Treatment for pneumonia using any terminology except with “hospital-acquired pneumonia” (HAP) or “ventilator-associated pneumonia” (VAP) were counted. Provider-selected indication of CAP (in an antibiotic order) was compared against this reference standard; sensitivity, specificity, and positive and negative predictive values were calculated. Performance characteristics of *International Classification of Disease, Tenth Revision* (ICD-10) codes for pneumonia in identifying CAP patients were assessed against the same reference standard. A secondary analysis including terms HAP and VAP in the reference standard was performed. **Results:** Provider-selected antibiotic indication for CAP had a sensitivity of 64.4%, specificity of 96.3%, positive predictive value (PPV) of 73.1%, and negative predictive value (NPV) of 96.1%, giving comparable performance to ICD-10 codes (Table 1). Of those with 21 false-negative results, 13 (61.9%) had a healthcare-associated lower respiratory tract infection and 14 (66.7%) had sepsis indicated in at least 1 antibiotic order. **Conclusions:** Provider-selected antibiotic indication showed moderate sensitivity and high specificity for identifying CAP-treated cases. Importantly, use of this method can be deployed for real-time antibiotic stewardship interventions for CAP.

**Figure:**
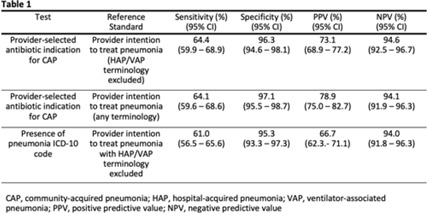

**Disclosures:** None

